# Correlation between lactate/albumin ratio and 28-day mortality in sepsis-associated acute kidney injury patients

**DOI:** 10.3389/fmed.2025.1546112

**Published:** 2025-06-05

**Authors:** Susu He, Yongkang Wang, Shiyu Wei, Shifeng Yang

**Affiliations:** ^1^Department of Nephrology, The First Affiliated Hospital of Xi’an Jiaotong University, Xi’an, China; ^2^Department of Hepatobiliary Surgery, Shaanxi Provincial People’s Hospital, Xi’an, China; ^3^Department of Hepatobiliary Surgery, The First Affiliated Hospital of Xi’an Jiaotong University, Xi’an, China

**Keywords:** sepsis-associated acute kidney injury, lactate/albumin ratio, mortality, 28-day, MIMIC-IV

## Abstract

**Background:**

The lactate/albumin ratio (LAR) was related to adverse outcomes in heart failure, myocardial infarction, and acute pancreatitis. However, it remains unevaluated whether LAR has prognosis significance in sepsis-associated acute kidney injury (SA-AKI) patients. Therefore, this research was performed to clarify the potential predictive utility of LAR for 28-day mortality in SA-AKI patients.

**Methods:**

Participants diagnosed with SA-AKI were selected from the Medical Information Mart for Intensive Care (MIMIC-IV) database and then placed into four groups in accordance with LAR quartiles. The endpoint was all-cause mortality within 28 days. The Kaplan–Meier curves were conducted to estimate the cumulative survival rates in the four groups. The correlation between LAR and the endpoint was elucidated by constructing Cox proportional hazards analysis, restricted cubic splines (RCS), and subgroup analysis.

**Results:**

Of the 3,895 patients with SA-AKI, 58.59% were men. The mortality rate from all causes within 28 days was 28.55%. The Kaplan–Meier curves showed that participants having elevated LAR exhibited significantly decreased survival rates. Cox proportional hazards models showed that higher LAR was related to higher 28-day death rate in SA-AKI patients (HR: 1.224, 95% CI: 1.160–1.291, *p* < 0.001). In addition, RCS analyses revealed that LAR was non-linearly correlated with the risk of 28-day death.

**Conclusion:**

LAR was independently related to an elevated risk of mortality within 28 days in SA-AKI patients. More prospective research studies are necessitated for further confirmation of these findings.

## Introduction

Sepsis represents a life-threatening circumstance that stems from the host’s abnormal reaction in the face of infection, leading to organ dysfunction with abnormal signs and laboratory evidence (1). The kidney is frequently affected, leading to the occurrence of sepsis-associated acute kidney injury (SA-AKI). Approximately 30–50% of acute kidney injury (AKI) that occur in the intensive care unit (ICU) are notably caused by sepsis (2–6). SA-AKI usually can contribute to prolonged hospital stays, elevated morbidity, and increased economic burdens for patients (7–11). However, due to the diverse and intricate underlying mechanisms of SA-AKI (12, 13), it is still a huge challenge to forecast SA-AKI patients’ clinical outcomes. Therefore, it is crucial to find valuable predictors to reduce adverse outcomes among patients with SA-AKI.

Lactate, which is produced during anaerobic metabolism as a secondary or by-product, can accurately reflect the extent of cellular hypoxia and tissue hypoperfusion (14). However, lactate may be elevated in individuals with hepatic dysfunction and diabetes, or those taking metformin (15, 16), which may make prediction solely based on lactate levels potentially unreliable.

In addition, serum albumin, which is considered as an important protein, plays a significant part in maintaining the balance of blood colloid osmotic pressure, endothelial stability, and immune regulation. Several research studies already revealed that albumin can forecast poor outcomes among patients with sepsis (17) or AKI (18). However, nutritional condition, inflammatory infection, and liver or kidney disease may influence albumin levels. Thus, depending merely on albumin levels in forecasting outcomes can also have limited value.

Previous studies already revealed that lactate/albumin ratio (LAR) is related to adverse outcomes in acute myocardial infarction, acute pancreatitis, and heart failure (19–24). Nevertheless, the link between LAR and SA-AKI patients’ prognosis is still not definite. Consequently, the research was performed to investigate the potential link between LAR in SA-AKI patients and 28-day mortality from all causes.

## Methods

### Study population

The data we analyzed were sourced from a freely accessible MIMIC-IV database, which encompasses the clinical records regarding patients hospitalized in ICU of the Beth Israel Deaconess Medical Center during the period from 2008 to 2019. It documents detailed records on patients’ demographics, vital signs, laboratory measures, medication treatment, disease diagnosis, medication management, and other comprehensive information (25). Because of the anonymization of patients’ health data in the database, ethics approval and informed consent were exempted. Our study confirmed compliance with the Declaration of Helsinki and MIMICIV’s data usage agreements. To get data access, we participated in the training course of the National Institutes of Health and successfully completed the exams of the Collaborative Institutional Training Initiative. Subsequently, we got the authorization to make use of the database and extract relevant data.

**Figure 1:**
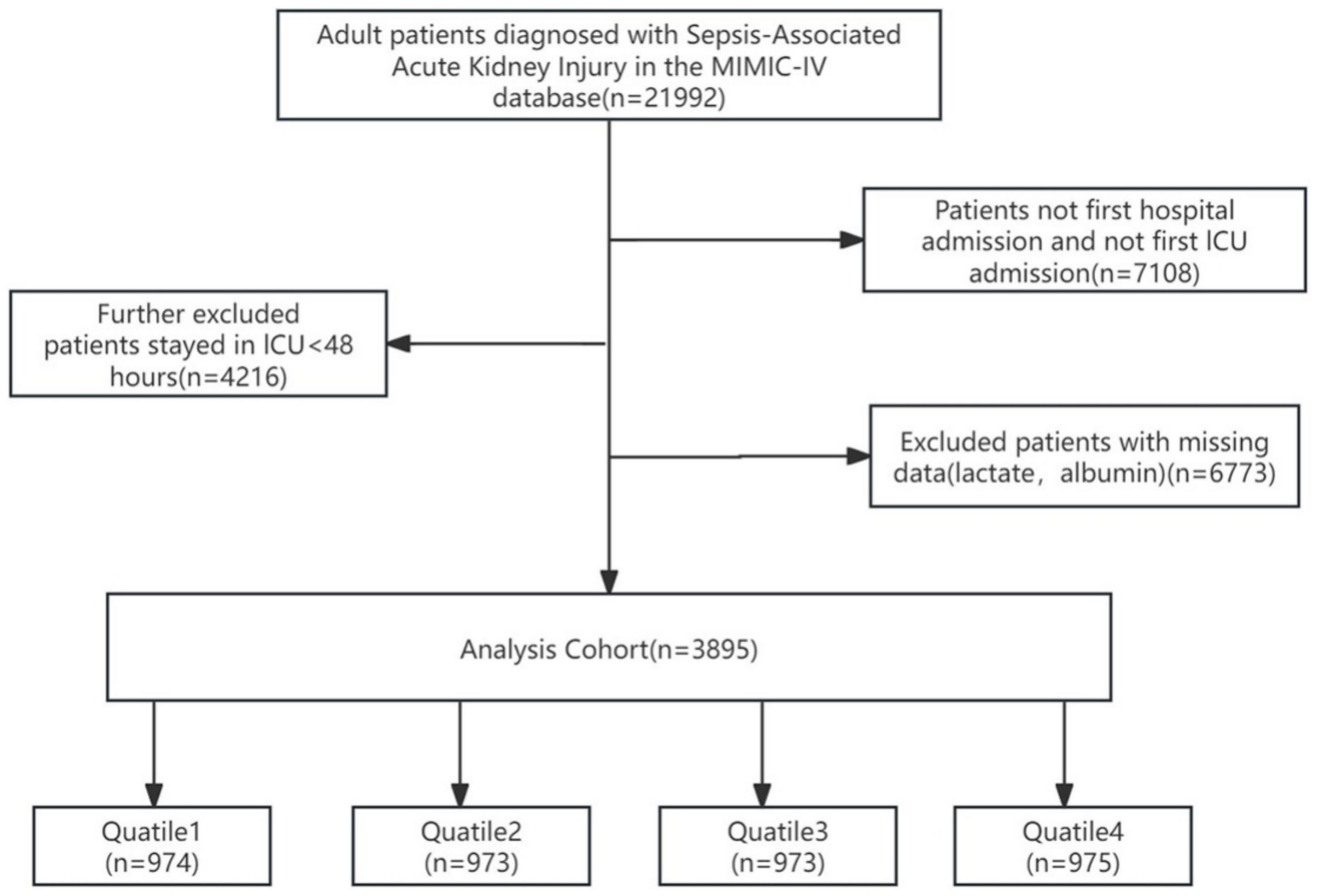
Patient selection process.

Patients admitted to the ICU with sepsis and developing AKI within 48 h were incorporated into this research. Sepsis was determined as an increase in the sequential organ failure assessment (SOFA) scale score by 2 points, accompanied by a confirmed or suspected infection (1). The definition of AKI conformed to the Kidney Disease: Improving Global Outcomes criteria, which both takes into account the highest serum creatinine value and urine output (26). The minimum value of SCr within the 7-day period preceding ICU admission was adopted as the baseline SCr. In case of the absence of SCr prior to admission, the initial SCr of the patient after ICU admission was regarded as the relevant one. Only measurement from the first ICU admission was employed for research in case of multiple admissions. First, we excluded participants with ICU duration shorter than 48 h to ensure adequate observation of SA-AKI progression and minimize potential confounding from incomplete disease manifestation or early discharge. Subsequently, we further excluded those participants in the following order: (1) participants with multiple ICU/hospital admissions with SA-AKI, using only the first admission examination; (2) participants lacking lactate and albumin data. In total, 3,895 participants were finally incorporated into the research (Figure 1).

We chose the initial blood lactate/serum albumin ratio upon admission within 24 h as the primary research variable to reduce the impact of subsequent clinical interventions. The variables relevant to this study were all obtained by structured query language. These variables included demographic details such as age, sex, and race, vital signs such as oxygen saturation (SpO2), systolic blood pressure (SBP), diastolic blood pressure (DBP), mean blood pressure (MBP), respiratory rate (RR), and heart rate (HR), laboratory examination data such as platelet, hemoglobin, white blood cell (WBC), hematocrit, sodium, potassium, calcium, anion gap, chloride, blood urea nitrogen (BUN), glucose, base excess, bicarbonate, and serum creatinine (SCr). Also encompassed were comorbidities such as liver disease, congestive heart failure (CHF), chronic pulmonary disease, malignancy, diabetes, myocardial infarction (MI), renal disease, disease severity scores upon admission such as the SOFA score and charlson comorbidity index (CCI), AKI stage, and the utilization of renal replacement therapy (RRT). All laboratory indicators were sourced from the initial ICU examination. The follow-up process commenced immediately upon the patients’ admission and continued until the time of their death. Variables missing over 20% of values, such as lymphocytes, monocytes, neutrophils, lactate dehydrogenase, globulin, height, and weight, were excluded. For those missing less than 20%, multiple imputation was utilized by the missForest package of R software to fill in the missing values (27).

### Primary outcome

The mortality from all causes within 28 days was the primary outcome.

### Statistical analysis

Based on the distribution status, normally distributed continuous variables were described in the form of mean ± standard deviation (SD) and analyzed using *t*-tests or ANOVA, while non-normally distributed continuous variables were described in the form of median with interquartile range (IQR) and evaluated through Mann–Whitney U tests or Kruskal–Wallis tests. For categorical variables, proportions were reported.

Kaplan–Meier (KM) survival curves were performed to calculate the cumulative survival rates in the four groups, with differences examined via the log-rank test. A univariate Cox analysis was conducted to determine possible confounders. The multivariate Cox proportional hazards regression model encompassed variables with clinical significance or *p* < 0.05 in the univariate analysis. Model 1 employed the unadjusted reference. In Model 2, adjustments for age, sex, and ethnicity were made. Model 3, building upon Model 2, accounted for factors such as HR, SpO_2_, SBP, DBP, hemoglobin, WBC, platelet, SCr, glucose, potassium, sodium, BUN, MI, CHF, malignancy, renal disease, diabetes, liver disease, RRT, SOFA score, and CCI. LAR was examined by employing restricted cubic splines (RCS) to explore its non-linear association with 28-day mortality. Receiver operating characteristic (ROC) analysis was utilized to assess the diagnostic capacity, sensitivity, and specificity of lactate, albumin, LAR, SOFA, and CCI for mortality with AUC calculated for each index. Finally, subgroup analysis was conducted to assess potential moderating effects according to age (≥ 65 or < 65 years), sex, ethnicity, MI, CHF, liver disease, diabetes, renal disease, and RRT.

R software (version 4.3.3) and SPSS software (version 26.0) were used to analyze the data, and a *p*-value of < 0.05 was considered significant for two-sided tests.

## Results

### Baseline characteristics

There were 3,895 participants with SA-AKI in total that were ultimately included. The patients’ median age at baseline was 66 (IQR: 54–77) years. There were 2,282 (58.59%) males. The median LAR of all included participants was 0.69 (0.44, 1.18), and the mortality rate within 28 days was 28.55%.

Table 1 exhibits the participants’ baseline characteristics that were grouped into four groups in accordance with LAR quartiles. The median value of LAR in each quartile was 0.34 (IQR: 0.28–0.39), 0.55 (IQR: 0.50–0.62), 0.88 (IQR: 0.78–1.01), and 1.81 (IQR: 1.45–2.50), respectively. In comparison with the lower group, those with higher LAR were younger and more prone to have liver disease and renal disease, had higher disease severity scores at admission and higher usage rate of RRT and had higher HR, RR, WBC, glucose, SCr, BUN, anion gap, base excess, potassium, and lactate and lower SBP, DBP, MBP, and SpO_2_. In addition, as LAR increased, the risk of mortality within 28 days increased (19.30% versus 26.41% versus 29.09% versus 39.38%, *p* < 0.001).

**Table 1:** Baseline characteristics of 3,895 participants by LAR quartiles.

Variables	Overall	Q1	Q2	Q3	Q4	*p*-value
Demographics						
Age (years)	66 (54, 77)	66 (54, 77)	67 (56, 79)	66 (54, 78)	64 (53, 75)	<0.001
Sex (%)						0.379
Female	1,613 (41.41)	419 (43.02)	385 (39.57)	413 (42.45)	396 (40.62)	
Male	2,282 (58.59)	555 (56.98)	588 (60.43)	560 (57.55)	579 (59.38)	
Ethnicity (%)						0.009
White	2,385 (61.23)	640 (65.71)	579 (59.51)	600 (61.66)	566 (58.05)	
Black	336 (8.63)	64 (6.57)	97 (9.97)	80 (8.22)	95 (9.74)	
Other	1,174 (30.14)	270 (27.72)	297 (30.52)	293 (30.11)	314 (32.21)	
Vital signs						
Heart rate (beats/min)	89 (77, 102)	84 (73, 95)	87 (76, 99)	91 (78, 103)	95 (83, 110)	<0.001
Respiratory rate (beats/min)	20.1 (17.5, 23.5)	19.5 (17.2, 22.3)	19.6 (17.3, 22.9)	20.3 (17.5, 23.8)	21.1 (18.3, 24.7)	<0.001
Oxygen saturation (%)	97.37 (95.77, 98.74)	97.08 (95.57, 98.67)	97.36 (95.77, 98.64)	97.58 (95.96, 98.82)	97.45 (95.80, 98.84)	0.012
Systolic blood pressure (mmHg)	111 (103, 121)	115 (107, 127)	112 (104, 123)	110 (103, 119)	108 (101, 115)	<0.001
Diastolic blood pressure (mmHg)	61 (54, 67)	61 (55, 69)	61 (55, 68)	60 (54, 67)	60 (53, 66)	<0.001
Mean blood pressure (mmHg)	75 (70, 82)	76 (70, 84)	76 (71, 83)	75 (69, 82)	74 (69, 80)	<0.001
Laboratory tests						
Lactate (mmol/L)	2.10 (1.40, 3.43)	1.10 (0.90, 1.30)	1.75 (1.50, 2.03)	2.60 (2.20, 3.17)	4.99 (3.86, 6.67)	<0.001
Albumin (g/dL)	3.10 (2.60, 3.60)	3.40 (3.00, 3.80)	3.20 (2.70, 3.65)	3.00 (2.55, 3.40)	2.65 (2.20, 3.10)	<0.001
Lactate/albumin ratio	0.69 (0.44, 1.18)	0.34 (0.28, 0.39)	0.55 (0.50, 0.62)	0.88 (0.78, 1.01)	1.82 (1.45, 2.50)	<0.001
Hematocrit (%)	32 (28, 37)	32 (28, 37)	33 (29, 38)	32 (28, 37)	31 (27, 35)	<0.001
Hemoglobin (g/dL)	10.55 (9.12, 12.20)	10.70 (9.20, 12.15)	10.90 (9.43, 12.64)	10.53 (9.11, 12.10)	10.10 (8.93, 11.70)	<0.001
Platelet (k/uL)	177 (116, 245)	203 (153, 268)	188 (139, 259)	166 (107, 239)	135 (91, 208)	<0.001
White blood cell (k/uL)	13 (9, 17)	11 (9, 15)	13 (10, 17)	13 (9, 18)	14 (9, 19)	<0.001
Glucose (mg/dL)	143 (116, 186)	128 (109, 162)	142 (117, 182)	152 (121, 194)	156 (122, 209)	<0.001
Serum creatinine (mg/dL)	1.28 (0.87, 2.16)	1.08 (0.75, 2.13)	1.20 (0.85, 2.10)	1.29 (0.90, 2.03)	1.60 (1.07, 2.40)	<0.001
Blood urea nitrogen (mg/dL)	26 (17, 43)	23 (15, 42)	26 (17, 44)	27 (18, 42)	27 (18, 43)	<0.001
Anion gap (mEq/L)	15.7 (13.2, 18.7)	14.5 (12.6, 16.8)	15.0 (13.0, 17.5)	15.5 (13.0, 18.3)	18.1 (15.3, 21.8)	<0.001
Base excess (mmol/L)	−2.3 (−6.0, 0.0)	0.0 (−3.0, 2.0)	−1.1 (−4.3, 0.9)	−2.7 (−5.6, −0.1)	−6.0 (−9.3, −2.9)	<0.001
Bicarbonate (mEq/L)	21.3 (18.2, 24.3)	23.4 (20.5, 26.0)	22.0 (19.5, 25.0)	21.0 (18.3, 23.6)	18.6 (16.0, 21.5)	<0.001
Chloride (mEq/L)	104 (100, 108)	104 (100, 108)	104 (100, 108)	105 (100, 108)	105 (100, 109)	0.007
Sodium (mEq/L)	138.6 (135.5, 141.3)	138.8 (136.0, 141.5)	138.6 (135.5, 141.0)	138.3 (135.0, 141.0)	139.0 (135.4, 142.0)	0.003
Potassium (mEq/L)	4.20 (3.87, 4.70)	4.15 (3.83, 4.60)	4.17 (3.85, 4.60)	4.27 (3.90, 4.77)	4.30 (3.90, 4.84)	<0.001
Calcium (mg/dl)	8.10 (7.60, 8.60)	8.30 (7.87, 8.70)	8.17 (7.65, 8.63)	8.00 (7.57, 8.55)	7.94 (7.43, 8.54)	<0.001
Scoring systems						
CCI	6.0 (4.0, 8.0)	6.0 (4.0, 8.0)	6.0 (4.0, 8.0)	6.0 (4.0, 8.0)	6.0 (4.0, 8.0)	0.030
SOFA	4.0 (2.5, 6.0)	3.0 (2.0, 4.0)	4.0 (2.0, 5.0)	4.0 (3.0, 6.0)	5.0 (3.0, 7.0)	<0.001
Comorbidities (%)						
Myocardial infarction	750 (19.26)	183 (18.79)	195 (20.04)	186 (19.12)	186 (19.08)	0.907
Congestive heart failure	1,263 (32.43)	365 (37.47)	338 (34.74)	297 (30.52)	263 (26.97)	<0.001
Chronic pulmonary disease	1,056 (27.11)	332 (34.09)	281 (28.88)	252 (25.90)	191 (19.59)	<0.001
Renal disease	903 (23.18)	256 (26.28)	225 (23.12)	221 (22.71)	201 (20.62)	0.029
Malignancy	552 (14.17)	116 (11.91)	106 (10.89)	156 (16.03)	174 (17.85)	<0.001
Liver disease	1,052 (27.01)	141 (14.48)	210 (21.58)	296 (30.42)	405 (41.54)	<0.001
Diabetes	1,214 (31.17)	316 (32.44)	306 (31.45)	301 (30.94)	291 (29.85)	0.661
Interventions						
RRT use (%)	675 (17.33)	113 (11.60)	120 (12.33)	148 (15.21)	294 (30.15)	<0.001
AKI stage (%)						
1	811 (20.82)	234 (24.02)	227 (23.33)	203 (20.86)	147 (15.08)	
2	1703 (43.72)	491 (50.41)	439 (45.12)	435 (44.71)	338 (34.67)	
3	1,381 (35.46)	249 (25.56)	307 (31.55)	335 (34.43)	490 (50.26)	
LOS						
Hospital LOS	13 (7, 21)	12 (7, 19)	12 (7, 20)	13 (8, 21)	14 (7, 24)	0.003
ICU LOS	6 (3, 11)	5 (3, 10)	6 (4, 10)	6 (3, 10)	7 (4, 12)	<0.001
Outcome						
28-day mortality (%)	1,112 (28.55)	188 (19.30)	257 (26.41)	283 (29.09)	384 (39.38)	<0.001

CCI (Charlson comorbidity index), RRT (renal replacement therapy), SOFA (sequential organ failure assessment), AKI (acute kidney injury), LOS (length of stay).

Table 2 exhibits the detailed baseline characteristics between the survival and non-survival groups. The participants in non-survival group were older (*p* < 0.001) and had higher incidence of CHF, liver disease, malignancy, higher disease severity scores at admission, and higher usage rate of RRT (*p* < 0.05). As for clinical laboratory data, non-survival group had higher HR, RR, WBC, glucose, SCr, BUN, anion gap, base excess, potassium, lactate (*p* < 0.05), LAR (0.85 [0.52, 1.58] vs. 0.64 [0.42, 1.06], *p* < 0.001) and lower SpO_2_, SBP, DBP, MBP, hemoglobin, platelet, albumin, and bicarbonate (*p* < 0.05).

**Table 2:** Baseline characteristics of 3,895 participants between two groups.

Variables	Overall (*n* = 3,895)	Survival group (*n* = 2,783)	Non-survival group (*n* = 1,112)	*p*-value
Demographics				
Age (years)	66 (54, 77)	64 (53, 75)	70 (57, 81)	<0.001
Sex (%)				0.773
Female	1,613 (41.41)	1,148 (41.25)	465 (41.82)	
Male	2,282 (58.59)	1,635 (58.75)	647 (58.18)	
Ethnicity (%)				<0.001
White	2,385 (61.23)	1776 (63.82)	609 (54.77)	
Black	336 (8.63)	254 (9.13)	82 (7.37)	
Other	1,174 (30.14)	753 (27.06)	421 (37.86)	
Vital signs				
Heart rate (beats/min)	89 (77, 102)	88 (77, 101)	91 (77, 104)	0.029
Respiratory rate (beats/min)	20.1 (17.5, 23.5)	19.8 (17.3, 23.1)	20.8 (18.1, 24.3)	<0.001
Oxygen saturation (%)	97.37 (95.77, 98.74)	97.42 (95.92, 98.76)	97.24 (95.35, 98.68)	0.003
Systolic blood pressure (mmHg)	111 (103, 121)	112 (104, 122)	110 (102, 119)	<0.001
Diastolic blood pressure (mmHg)	61 (55, 67)	61 (55, 68)	60 (53, 67)	0.002
Mean blood pressure (mmHg)	75 (70, 82)	76 (70, 82)	74 (68, 81)	<0.001
Laboratory tests				
Lactate (mmol/L)	2.10 (1.40, 3.43)	1.97 (1.30, 3.20)	2.45 (1.60, 4.23)	<0.001
Albumin (g/dL)	3.10 (2.60, 3.60)	3.10 (2.65, 3.60)	3.00 (2.45, 3.50)	<0.001
Lactate/albumin ratio	0.69 (0.44, 1.18)	0.64 (0.42, 1.06)	0.85 (0.52, 1.58)	<0.001
Hematocrit (%)	32 (28, 37)	32 (28, 37)	32 (27, 37)	0.095
Hemoglobin (g/dL)	10.55 (9.12, 12.20)	10.63 (9.24, 12.30)	10.33 (8.90, 12.02)	<0.001
Platelet (k/Ul)	177 (116, 245)	178 (120, 246)	173 (106, 245)	0.023
White blood cell (k/uL)	13 (9, 17)	12 (9, 17)	14 (10, 19)	<0.001
Glucose (mmol/L)	143 (116, 186)	141 (116, 183)	148 (117, 192)	0.014
Serum creatinine (mg/dL)	1.28 (0.87, 2.16)	1.20 (0.85, 2.05)	1.50 (0.97, 2.37)	<0.001
Blood urea nitrogen (mg/dL)	26 (17, 43)	24 (16, 39)	31 (20, 50)	<0.001
Anion gap (mEq/L)	15.7 (13.2, 18.7)	15.3 (13.0, 18.0)	16.7 (14.0, 20.0)	<0.001
Base excess (mmol/L)	−2.3 (−6.0, 0.0)	−2.0 (−5.5, 0.2)	−3.4 (−7.5, 0.0)	<0.001
Bicarbonate (mEq/L)	21.3 (18.2, 24.3)	21.6 (18.7, 24.5)	20.4 (17.2, 23.7)	<0.001
Chloride (mEq/L)	104 (100, 108)	104 (101, 108)	104 (99, 108)	0.194
Sodium (mg/dL)	138.6 (135.5, 141.3)	138.5 (135.8, 141.0)	139.0 (135.0, 142.5)	0.053
Potassium (mg/dL)	4.20 (3.87, 4.70)	4.20 (3.85, 4.66)	4.28 (3.88, 4.80)	0.001
Calcium (mg/dL)	8.10 (7.60, 8.60)	8.10 (7.63, 8.60)	8.13 (7.56, 8.65)	0.963
Scoring systems				
CCI	6.0 (4.0, 8.0)	6.0 (4.0, 8.0)	7.0 (5.0, 9.0)	<0.001
SOFA	4.0 (2.5, 6.0)	4.0 (2.0, 5.0)	4.0 (3.0, 6.0)	<0.001
Comorbidities (%)				
Myocardial infarction	750 (19.26)	515 (18.50)	235 (21.13)	0.067
Congestive heart failure	1,263 (32.43)	876 (31.48)	387 (34.80)	0.049
Chronic pulmonary disease	1,056 (27.11)	752 (27.02)	304 (27.34)	0.872
Liver disease	1,052 (27.01)	691 (24.83)	361 (32.46)	<0.001
Diabetes	1,214 (31.17)	890 (31.98)	324 (29.14)	0.091
Renal disease	903 (23.18)	634 (22.78)	269 (24.19)	0.368
Malignancy	552 (14.17)	333 (11.97)	219 (19.69)	<0.001
Interventions				
RRT use (%)	675 (17.33)	413 (14.84)	262 (23.56)	<0.001
AKI stage (%)				
1	811 (20.82)	640 (23.00)	171 (15.38)	
2	1703 (43.72)	1,295 (46.53)	408 (36.70)	
3	1,381 (35.46)	848 (30.47)	533 (47.93)	

CCI (Charlson comorbidity index), RRT (renal replacement therapy), SOFA (sequential organ failure assessment), AKI (acute kidney injury).

### Kaplan–Meier survival curves

**Figure 2:**
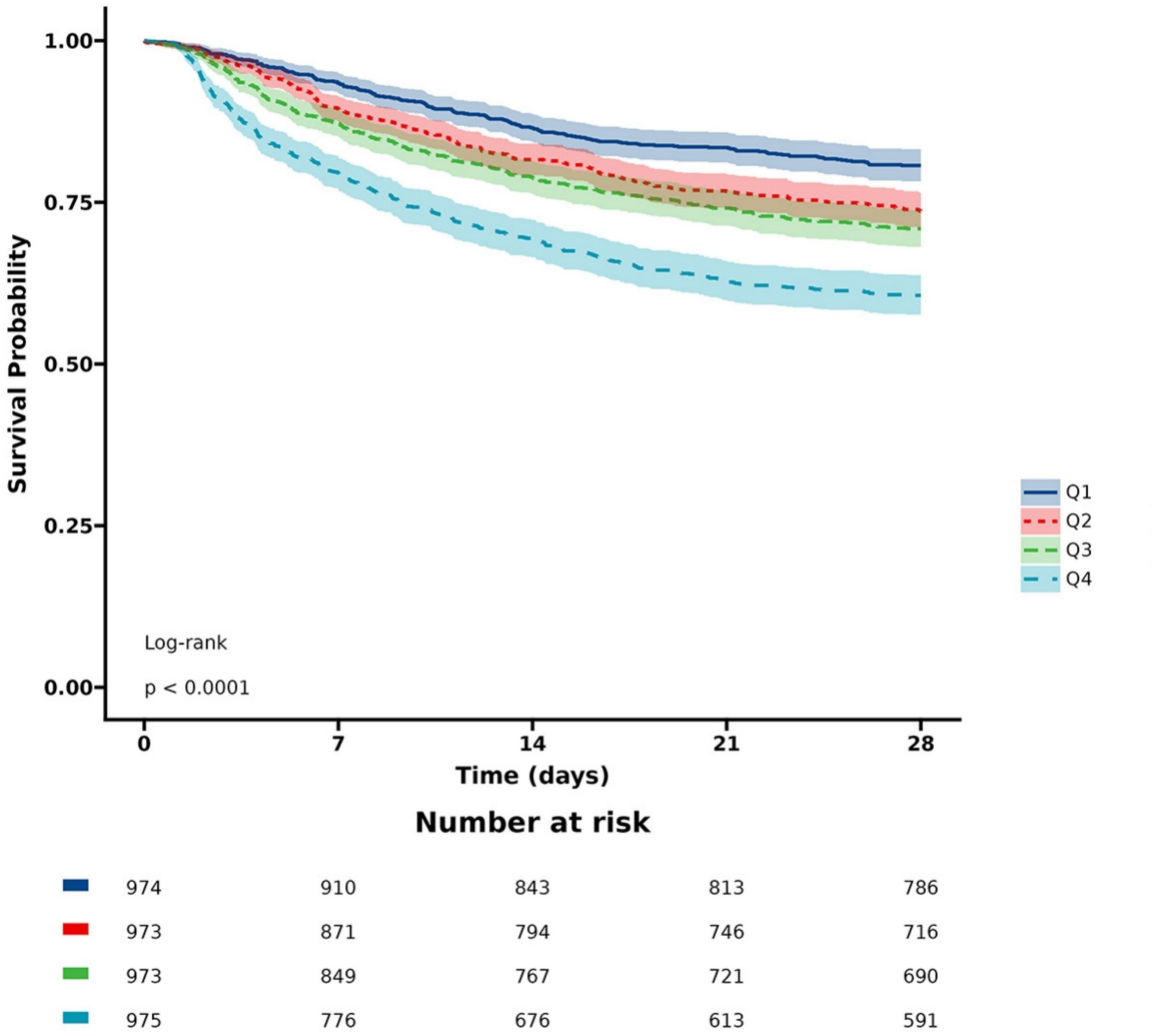
KM curves for 28-day mortality. LAR: Q1 (0.08–0.44), Q2 (0.44–0.69), Q3 (0.69–1.18), and Q4 (1.18–10.15).

Figure 2 exhibits the cumulative survival rates in the four groups in accordance with LAR quartiles. Participants possessing a higher LAR showed a considerably poorer survival rate in comparison with those having a lower LAR (log-rank *p* < 0.001).

### The correlation between LAR and primary outcome

Subsequent to the univariate Cox regression analysis (detailed in Supplementary Table S1), three disparate multivariate models were constructed and applied to estimate the relation between LAR and the primary outcome. The analysis manifested that when LAR was regarded as a continuous variable, it had a strong relation with mortality risk within 28 days in three models (Model 1: HR: 1.327, 95% CI: 1.270–1.387, *p* < 0.001, Model 2: HR: 1.320, 95% CI: 1.264–1.379, *p* < 0.001, Model 3: HR: 1.224, 95% CI: 1.160–1.291, *p* < 0.001). When LAR was regarded as a nominal variable, the patients in the highest quartile demonstrated the highest 28-day death rate in the three established models (Model 1: HR: 2.412, 95% CI: 2.026–2.872, *p* < 0.001, Model 2: HR: 2.459, 95% CI: 2.064–2.929, *p* < 0.001, Model 3: HR: 1.837, 95% CI: 1.513–2.232, *p* < 0.001) (Table 3).

**Table 3:** Multivariate Cox proportional hazards models of 28-day mortality.

Categories	Model 1		Model 2		Model 3	
	HR (95% CI)	*p*-value	HR (95% CI)	*p*-value	HR (95% CI)	*p*-value
LAR as continuous	1.327 (1.270, 1.387)	<0.001	1.320 (1.264, 1.379)	<0.001	1.224 (1.160, 1.291)	<0.001
Quartile						
Q1	Ref (1)		Ref (1)		Ref (1)	
Q2	1.425 (1.181, 1.720)	<0.001	1.364 (1.130, 1.648)	0.001	1.245 (1.029, 1.508)	0.025
Q3	1.616 (1.344, 1.943)	<0.001	1.575 (1.310, 1.895)	<0.001	1.339 (1.105, 1.624)	0.003
Q4	2.412 (2.026, 2.872)	<0.001	2.459 (2.064, 2.929)	<0.001	1.837 (1.513, 2.232)	<0.001
*p* for trend		<0.001		<0.001		<0.001

Model 1: adjusted for no covariates. Model 2: adjusted for age, sex, and ethnicity. Model 3: on the basis of model 2 and further adjusted for HR, SpO_2_, SBP, DBP, hemoglobin, WBC, platelet, SCr, BUN, glucose, potassium, sodium, MI, CHF, malignancy, renal disease, diabetes, liver disease, RRT, SOFA score, and CCI.

### ROC curve analysis

**Figure 3:**
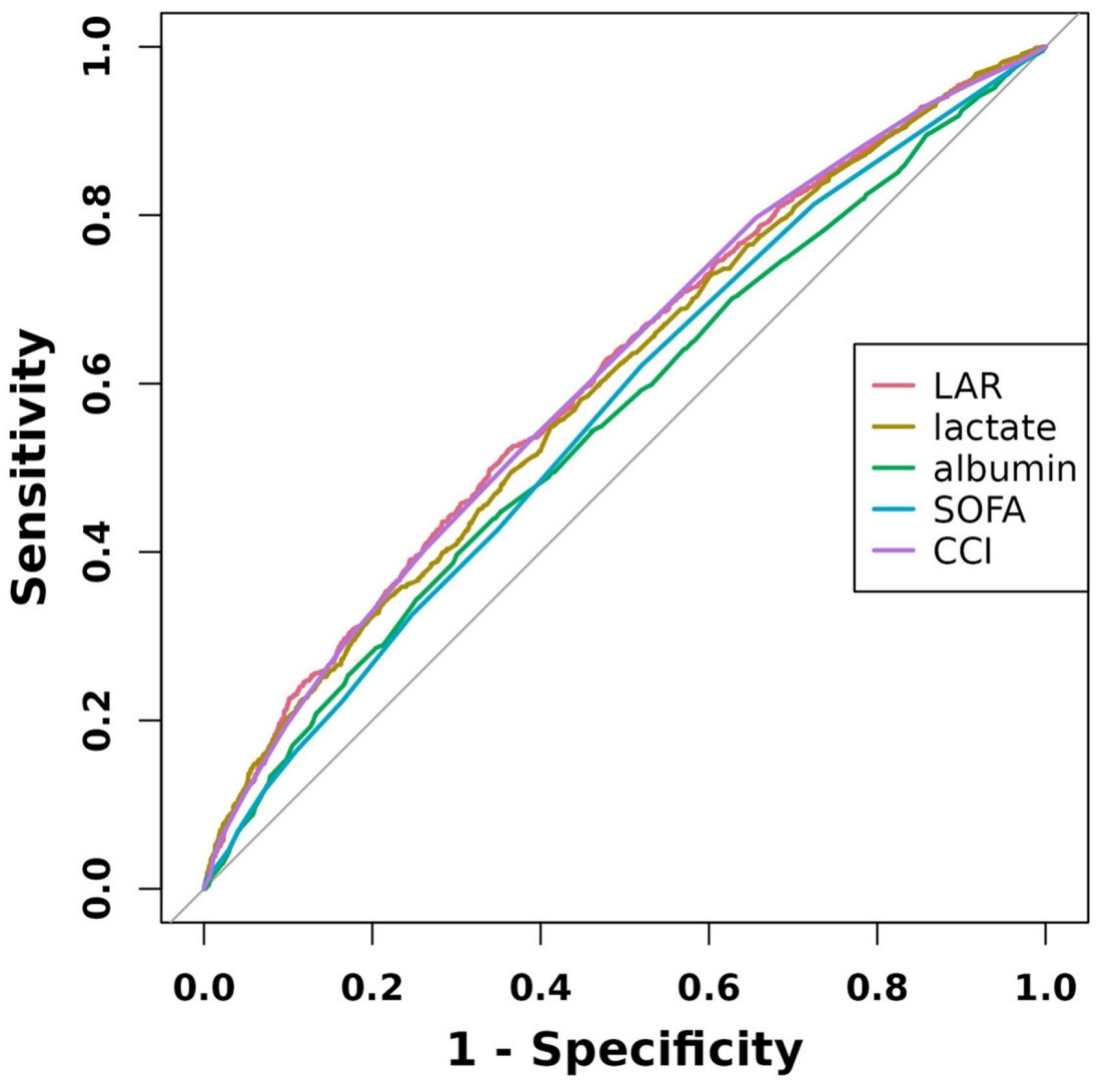
ROC curves of five indicators associated with 28-day mortality.

We plotted the ROC curves of five biomarkers (LAR, lactate, albumin, SOFA, and CCI) to estimate their predictive capability for mortality. As detailed in Table 4 and depicted in Figure 3, LAR’s AUC (0.6092, 95% CI: 0.5896–0.6288) was the highest in comparison with the other four indicators, including lactate (0.5982, 95% CI: 0.5785–0.6179), albumin (0.5584, 95% CI: 0.5382–0.5786), SOFA (0.5671, 95% CI: 0.5474–0.5869), and CCI (0.6089, 95% CI: 0.5895–0.6283). Therefore, LAR has a relatively better predictive performance. The results revealed that LAR exhibited a moderate diagnostic capacity of 28-day mortality. The ideal cutoff value was 0.83 with specificity of 63.7% and sensitivity of 52.2%.

**Table 4:** Details regarding ROC curves.

Variables	AUC	95% CI	Cutoff value	Sensitivity	Specificity
Lactate/albumin ratio	0.6092	0.5896–0.6288	0.83	0.522	0.637
Lactate	0.5982	0.5785–0.6179	2.29	0.549	0.587
Albumin	0.5584	0.5382–0.5786	2.76	0.397	0.699
Sequential organ failure assessment	0.5671	0.5474–0.5869	3.50	0.621	0.480
Charlson comorbidity index	0.6089	0.5895–0.6283	6.50	0.528	0.617

### Analyses of the non-linear relationship

**Figure 4:**
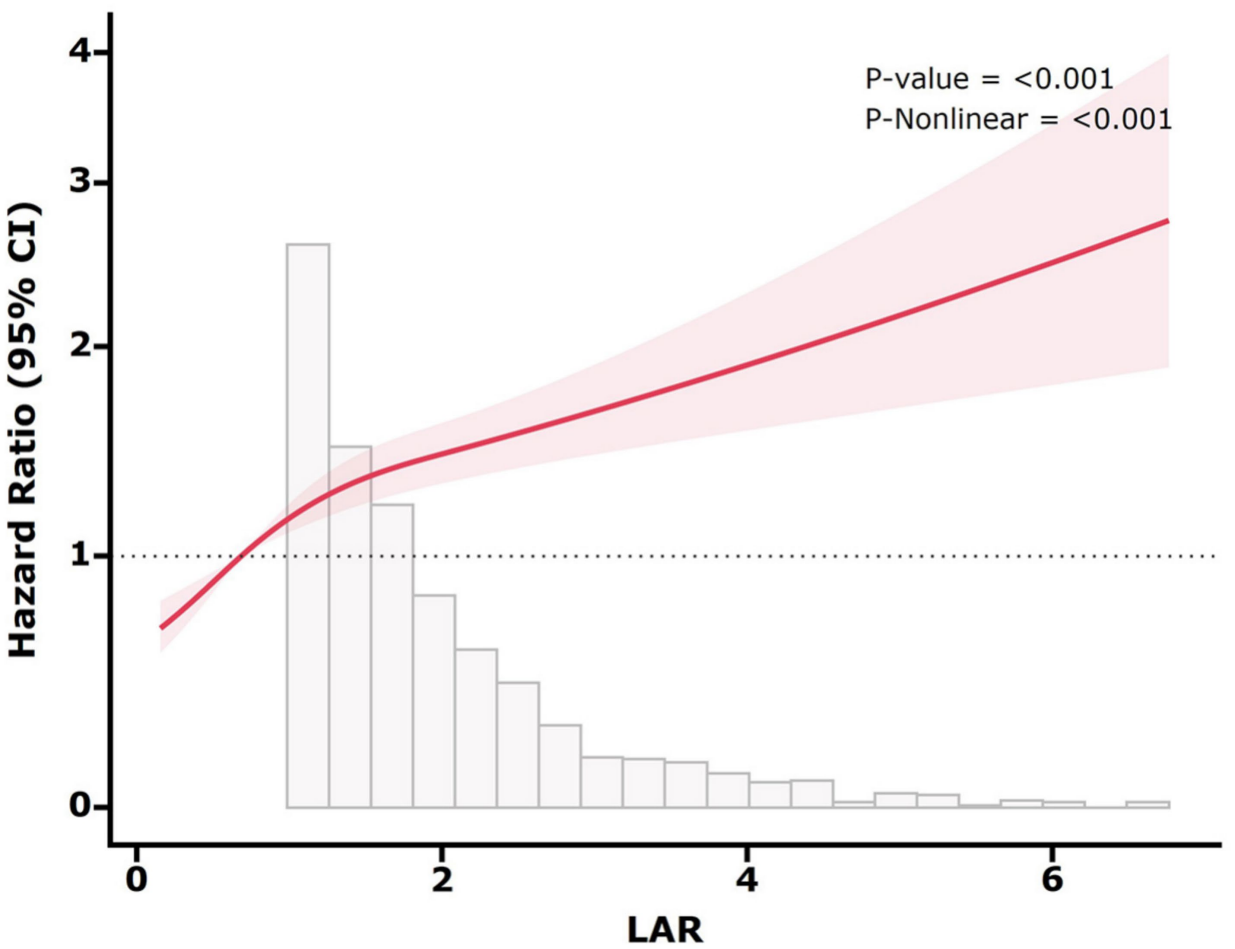
Restricted cubic spline for 28-day all-cause mortality. The red solid line represents the HR, and the shaded band represents the 95% CI. The model adjusted for age, sex, ethnicity, HR, SBP, DBP, SpO_2_, hemoglobin, platelet, WBC, BUN, SCr, glucose, sodium, potassium, MI, CHF, liver disease, renal disease, diabetes, malignancy, RRT, CCI, and SOFA.

We observed the non-linear relation between LAR and the mortality using restricted cubic splines (*p*-non-linear < 0.05) (Figure 4). Subsequently, we analyzed the influence of LAR levels on the all-cause death rate within 28 days based on a two-part Cox proportional hazards model. An inflection point was identified at 0.30. When LAR was less than 0.30, no statistically significant link between LAR and the mortality was discovered (*p* > 0.05). However, when LAR exceeded 0.30, it was markedly and positively correlated with the risk of 28-day death. With every 1-unit increase in LAR, there was a 28% greater risk of 28-day mortality (Table 5).

**Table 5:** Impact of LAR level on 28-day mortality based on the two-part Cox proportional hazard model.

LAR	HR (95% CI)	*p*-value
LAR (<0.3)	1.08 (0.81–1.44)	0.59
LAR (≥0.3)	1.28 (1.23–1.34)	<0.001

### Subgroup analysis

The relationship between LAR and SA-AKI was stratified according to age, sex, ethnicity, MI, CHF, liver disease, renal disease, diabetes, and RRT (Table 6). We observed an interaction in the age and renal disease subgroups. Nevertheless, there was no interaction in the sex, ethnicity, MI, CHF, liver disease, diabetes, and RRT subgroups.

**Table 6:** Subgroup analyses for the relationship of LAR with 28-day mortality.

Subgroup	Total	Case (%)	HR (95% CI)	*p*-value	*p* for interaction
Age					<0.001
≤65	1878	433 (23.06%)	1.377 (1.270–1.492)	<0.001	
>65	2017	679 (33.67%)	1.135 (1.050–1.226)	0.001	
Sex					0.876
Female	1,613	465 (28.83%)	1.194 (1.100–1.295)	<0.001	
Male	2,282	647 (28.35%)	1.253 (1.164–1.348)	<0.001	
Ethnicity					0.494
White	2,385	609 (25.53%)	1.255 (1.159–1.359)	<0.001	
Black	336	82 (24.40%)	1.317 (1.090–1.591)	0.004	
Other	1,174	421 (35.86%)	1.205 (1.105–1.314)	<0.001	
MI					0.832
No	3,145	877 (27.89%)	1.237 (1.165–1.313)	<0.001	
Yes	750	235 (31.33%)	1.136 (1.000–1.291)	0.051	
CHF					0.358
No	2,632	725 (27.55%)	1.254 (1.178–1.334)	<0.001	
Yes	1,263	387 (30.64%)	1.164 (1.045–1.297)	0.006	
Liver disease					0.405
No	2,843	751 (26.42%)	1.177 (1.090–1.271)	<0.001	
Yes	1,052	361 (34.32%)	1.308 (1.207–1.416)	<0.001	
Diabetes					0.340
No	2,681	788 (29.39%)	1.262 (1.183–1.348)	<0.001	
Yes	1,214	324 (26.69%)	1.147 (1.037–1.268)	0.008	
Renal disease					0.041
No	2,992	843 (28.18%)	1.259 (1.187–1.336)	<0.001	
Yes	903	269 (29.79%)	1.095 (0.948–1.266)	0.219	
RRT					0.468
No	3,220	850 (26.40%)	1.231 (1.147–1.321)	<0.001	
Yes	675	262 (38.81%)	1.216 (1.113–1.328)	<0.001	

MI (myocardial infarction), CHF (congestive heart failure), RRT (renal replacement therapy).

## Discussion

In the present research, we observed a strong relation between LAR and 28-day mortality in SA-AKI patients. Even following confounding factor adjustment, the conclusion still held. Therefore, LAR may have the potential to be an independent indicator that can influence the prognosis and management decisions in patients with SA-AKI.

Lactate serves as a significant indicator for blood perfusion, tissue oxygenation, and metabolism in the body (28). Hyperlactacidemia occurs in the situation of an imbalance between lactic acid production and metabolism (29). Recent investigations have found that elevated lactate levels are related to adverse outcomes among septic shock patients and extremely high mortality in ICU (30, 31). Our study found that in contrast to the survival cohort, the lactate levels in the non-survival group were significantly higher (*p* < 0.001). However, the fluctuations in lactate levels, which can be caused by multiple factors, are thereby rather intricate. For instance, among individuals suffering from liver diseases, lactate metabolism disorder is a common phenomenon (15). In addition, some drugs such as metformin or salbutamol can lead to abnormal elevations of lactate (16). Furthermore, in certain individuals with critical condition, it is particularly noteworthy that they may exhibit significantly lower lactate levels (32). Thus, merely using lactate for forecasting is inaccurate.

Serum albumin, as a negative acute phase reactant, is capable of reflecting the inflammatory level and systemic nutritional status. It performs a vital part in facilitating the transport of hormones and drugs, maintaining plasma oncotic pressure, and acting as a pH buffer. Recent research studies have discovered that the albumin level is strongly related to the adverse outcomes of sepsis patients (33, 34). A previous investigation already demonstrated a significant relation between hypoproteinemia and the poor prognosis in AKI patients (35). However, since chronic disease states, nutritional intervention measures, and inflammatory responses can influence the levels of serum albumin (36), relying solely on albumin may have some limitations. In this study, we used LAR to increase the precision in predicting the prognosis among SA-AKI patients. By means of the reverse changes from the two distinct mechanisms, the influence of an individual element on the modulatory mechanism was reduced.

LAR, a comprehensive indicator, simultaneously takes into account the inflammatory and nutritional condition and has become an important biomarker for forecasting the prognosis of diverse diseases. Some research studies have found that increased LAR is related to the development of multiple organ dysfunction syndrome and increased risk of mortality among patients suffering from severe sepsis and septic shock (37, 38). It is noteworthy that Michael et al. suggested using LAR as a better prognostic factor to stratify patients with sepsis in accordance with disease severity (39). In addition, LAR was related to poor prognosis in heart failure, myocardial infarction, and acute pancreatitis (19–24). Therefore, LAR can be applied as a potent clinical indicator for forecasting the outcome in SA-AKI patients. Analyzing the database, our study found a significant relation between an elevated LAR and a higher 28-day death rate in SA-AKI patients.

The RCS analysis demonstrated a non-linear association between LAR and 28-day mortality in SA-AKI patients, with an inflection point at 0.30. Although the precise biological mechanisms remain to be elucidated, LAR levels below 0.30 may reflect a compensatory state in which homeostatic mechanisms partially counteract the detrimental effects of sepsis and renal injury. In contrast, LAR exceeding this threshold suggests severe physiological derangement, characterized by impaired tissue oxygenation and microcirculatory dysfunction, thereby increasing the mortality rate. Clinically, this inflection point serves as a critical prognostic indicator. Physicians should consider intensified monitoring and early intervention when LAR approaches or surpasses 0.30. Therefore, our findings could enhance the utility of LAR in risk stratification and clinical decision-making for SA-AKI patients.

The subgroup analysis indicated that the prognostic capacity of LAR was more significant in the subgroups of patients without renal disease and aged ≤ 65 years (*p* for interaction < 0.05). Our study revealed that patients without underlying diseases (including MI, CHF, diabetes, and renal disease) had higher mortality risk within 28 days. This might be due to reverse causality. That is to say, patients with these diseases are given more intensive treatment. Meanwhile, abnormal LAR in these patients is corrected more promptly. In addition, LAR had a significant relation with higher mortality risk within 28 days in patients without renal disease. The reason for this inconsistent result may be that patients with renal disease are more likely to have adequate renal perfusion before SA-AKI occurs. Another interesting finding was that the prognostic capacity of LAR was extremely prominent in patients aged 65 years or younger. In contrast to the general perception, clinicians may focus more on elderly patients because of their greater prevalence of comorbidities. Nevertheless, our study advocated for giving equal attention to young patients as their mortality rate may be higher.

Cakir et al. demonstrated that LAR (AUC = 0.869) was better at predicting in-hospital mortality than albumin (AUC = 0.812) and lactate (AUC = 0.816) separately (40). Shin et al. reported that among 946 patients diagnosed with sepsis, LAR exhibited a better predictive capacity for 28-day mortality compared to lactate (41). Yoo et al. reported that LAR (AUC = 0.715) was better at forecasting mortality rate within 28 days compared to SOFA score (AUC = 0.669) in patients with sepsis and septic shock (42). These outcomes are in line with the conclusion of our research. Therefore, our findings may assist clinicians to make a more accurate assessment of the severity and prognosis for SA-AKI, allowing for earlier intervention and better management of patients. However, it is important to note that while LAR showed predictive potential for 28-day mortality in SA-AKI patients, its discriminative performance remained moderate. Consequently, LAR should be utilized as part of a comprehensive evaluation rather than a sole factor for clinical decision-making.

The mechanism explaining the correlation between LAR and SA-AKI has not been fully elucidated. During SA-AKI, the systemic inflammatory response caused by sepsis can cause microcirculatory disorders, resulting in insufficient perfusion of kidney, and a large amount of pyruvate is converted into lactate. Meanwhile, sepsis can directly damage cell mitochondria and affect the oxidative phosphorylation function, leading to metabolism shifts from oxidative phosphorylation to aerobic glycolysis, thereby promoting the generation of lactate (43). Albumin maintains renal function by maintaining hemodynamic stability, exerting an antioxidant effect, binding harmful substances, and regulating inflammation (44). Moreover, albumin can contribute to promoting the synthesis of DNA in renal tubular cells through a specific signaling pathway associated with Ca2+ (45). Individuals diagnosed with SA-AKI usually have a decrease in serum albumin levels. This is attributed to damage to the glomerular filtration membrane and increased permeability and inflammatory responses. Consequently, LAR may have potential benefits in forecasting the prognosis of SA-AKI patients as it combines the reverse changes caused by the different mechanisms of two independent predictors.

However, it is essential to recognize the limitations of the research. First, this retrospective investigation depended on observational research, and thus, a definitive causal relationship could not be established. Second, despite the adjustment for confounding variables, potential residual confounding factors could still affect the findings. Third, using multiple imputation for variables with less than 20% missing values may also introduce additional uncertainty and potentially affect the accuracy of our conclusion. Finally, the study solely concentrated on assessing baseline LAR. Further research is required to verify whether changes in LAR can also predict mortality. Therefore, future research efforts should consider using prospective or randomized controlled trials to determine causality and broaden the research scope, thereby verifying and strengthening the obtained findings.

## Conclusion

In summary, LAR can be applied as a potential predictor for 28-day all-cause mortality in those with SA-AKI. Monitoring LAR could contribute to treatment decisions, disease management, and prognostic assessment in clinical practice. Future research is necessary to investigate whether better control of LAR can lead to improved clinical outcomes.

## Data Availability

The raw data supporting the conclusions of this article will be made available by the authors, without undue reservation.
