# Supplementary material for: Correlation between lactate/albumin ratio and 28-day mortality in sepsis-associated acute kidney injury patients
Source: Front Med (Lausanne). 2025 Jun 5;12:1546112. doi: 10.3389/fmed.2025.1546112 (PMC12176584; doi:10.3389/fmed.2025.1546112)
Supplement: Supplementary file 1 [file Table_1.docx]

Table S1 Univariate Cox regression analysis for 28-day all-cause mortality

| **Variables** | **HR(95% CI)** | ***p*-value** |
| --- | --- | --- |
| Age | 1.018 (1.014,1.022) | ＜0.001 |
| Sex | 0.989 (0.878,1.114) | 0.850 |
| Ethnicity (White) | 1 (Ref) |  |
| Ethnicity (Black) | 0.953 (0.757,1.200) | 0.682 |
| Ethnicity (Other) | 1.546 (1.365,1.751) | ＜0.001 |
| HR | 1.004 (1.000,1.007) | 0.031 |
| SBP | 0.991（0.987,0.995） | ＜0.001 |
| DBP | 0.991（0.986,0.997） | 0.004 |
| SpO_2_ | 0.945（0.925,0.965） | ＜0.001 |
| Hematocrit | 0.995（0.986,1.005） | 0.319 |
| WBC | 1.015（1.010,1.020） | ＜0.001 |
| Hb | 0.966（0.939,0.993） | 0.014 |
| PLT | 1.000（0.999,1.000） | 0.254 |
| SCr | 1.023（0.991,1.056） | 0.160 |
| BUN | 1.008（1.006,1.010） | ＜0.001 |
| Potassium | 1.199（1.098,1.310） | ＜0.001 |
| Sodium | 1.012（1.001,1.023） | 0.037 |
| Calcium | 0.999（0.934,1.069） | 0.979 |
| Glucose | 1.001（1.000,1.002） | 0.002 |
| Chloride | 0.996（0.987,1.004） | 0.337 |
| LAR | 1.327（1.270,1.387） | ＜0.001 |
| RRT | 1.585（1.380,1.821） | ＜0.001 |
| MI | 1.158（1.002,1.337） | 0.046 |
| CHF | 1.120（0.990,1.267） | 0.071 |
| Chronic pulmonary disease | 1.009（0.885,1.152） | 0.890 |
| Diabetes | 0.886（0.778,1.008） | 0.067 |
| Liver disease | 1.348（1.189,1.528） | ＜0.001 |
| Renal disease | 1.059（0.923,1.215） | 0.414 |
| SOFA | 1.071（1.050,1.093） | ＜0.001 |
| CCI | 1.112（1.090,1.134） | ＜0.001 |

Abbreviations: HR, heart rate; SBP, systolic blood pressure; DBP, diastolic blood pressure; SpO_2_, oxygen saturation; WBC, white blood cell; Hb, hemoglobin; PLT, platelet; SCr, serum creatinine; BUN, blood urea nitrogen; LAR, lactate/albumin ratio; RRT, renal replacement therapy; MI, myocardial infarction; CHF, congestive heart failure; SOFA, sequential organ failure assessment; CCI, charlson comorbidity index.
